# Harnessing noncoding RNA‐based macrophage polarization: Emerging therapeutic opportunities for fibrosis

**DOI:** 10.1002/iid3.341

**Published:** 2020-10-20

**Authors:** Dexi Zhou, Yilai Wu, Sheng Wang, Jun Li, Jiajie Luan

**Affiliations:** ^1^ Department of Pharmacy Yijishan Hospital of Wannan Medical College Wuhu Anhui Province China; ^2^ School of Pharmacy Wannan Medical College Wuhu Anhui Province China; ^3^ Key Laboratory of Non‐Coding RNA Transformation Research of Anhui Higher Education Institution Wannan Medical College Wuhu Anhui Province China; ^4^ School of Pharmacy Anhui Medical University Hefei Anhui Province China

**Keywords:** circular RNA, fibrosis, long noncoding RNA, macrophage polarization, microRNA

## Abstract

**Aim:**

Organ fibrosis is a common pathological outcome of persistent tissue injury correlated with organ failure and death. Although current antifibrotic therapies have led to unprecedented successes, only a minority of patients with fibrosis benefit from these treatments. There is an urgent need to identify new targets and biomarkers that could be exploited in the diagnosis and treatment of fibrosis.

**Methods:**

Macrophages play a dual role in the fibrogenesis across different organs either by promoting pro‐inflammatory or anti‐inflammatory responses. Noncoding RNAs (ncRNAs) have been demonstrated to play key roles in macrophage functions by manipulating macrophage polarization. Therefore, understanding the mechanism of ncRNA‐associated macrophage polarization is important to move toward therapeutic interventions.

**Results:**

In this review, we provide an overview of recent insights into the role of ncRNAs in different fibrotic diseases by modulating macrophage phenotypic plasticity and functional heterogeneity. We also discuss the potential mechanisms of different ncRNAs integrate heterogeneous macrophages in fibrogenesis,including regulatory signatures, networks, and reciprocal interactions.

**Conclusions:**

A broader understanding of how ncRNA‐directed macrophage phenotype transition in immunity and fibrosis might promote the development of a novel strategy for antifibrotic treatment.

## Graphical abstract

Macrophages play a dual role in the fibrogenesis across different organs either by promoting pro‐inflammatory or anti‐inflammatory responses. Noncoding RNAs have been demonstrated to play key roles in macrophage functions by manipulating macrophage polarization.

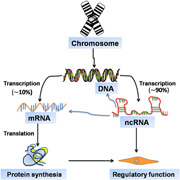

**Abbreviations**
AKI: acute kidney injury
ALD: alcoholic liver diseases
ALF: acute liver failure
ALI: acute lung injury
AM: alveolar macrophage
BM‐MSC: bone marrow‐derived mesenchymal stem cell
CAD: coronary artery disease
ceRNA: competing endogenous RNA
CF: cardiac fibrosis
CHB: chronic hepatitis B
COPD: chronic obstructive pulmonary disease
circRNA: circular RNA
CKD: chronic kidney diseases
CM: cardiac macrophages
CS: cigarette smoke
DN: diabetic nephropathy
DNMT3b: DNA methyltransferase 3b
DSS: dextran sulfate sodium
ECM: extracellular matrix
EMT: epithelial mesenchymal transition
EV: extracellular vesicle
HCC: hepatocellular carcinoma
HF: hepatic fibrosis
HSC: hepatic stellate cell
IFN‐γ: interferon‐γ
IL: interleukin
IM: infiltrated macrophage
IR: ischemia‐reperfusion
IRAK: IL‐1 receptor‐associated kinase
KC: Kupffer cell
lncRNA: long noncoding RNA
LXR: liver X receptor
MCD: methionine‐choline–deficient
MI: myocardial infarction
miRNA: microRNA
NASH: nonalcoholic steatohepatitis
ncRNA: noncoding RNA
NF‐κB: nuclear factor kappa B
NLRP3: nucleotide‐binding and oligomerization domain‐like receptor 3
NSCLC: non–small‐cell lung cancer
OSA: obstructive sleep apnea
PCG: protein‐coding gene
PF: pulmonary fibrosis
PPAR: peroxisome proliferator‐activated receptor
RF: renal fibrosis
RIF: renal interstitial fibrosis
RILF: radiation‐induced lung fibrosis
RM: renal macrophage
RNA‐Seq: RNA sequencing
SphK: sphingosine kinase
STAT1: signal transducer and activator of transcription 1
T‐UCR: transcribed ultraconserved region
TAM: tumor‐associated macrophage
TEC: tubular epithelial cell
TGF: transforming growth factor
TLR: toll‐like receptor
TMAO: trimethylamine *N*‐oxide
TNF‐α: tumor necrosis factor α
UTR: untranslated region

## 1 INTRODUCTION

Organ fibrosis is characterized by excessive deposition of connective tissue components and is commonly associated with high morbidity and mortality worldwide. Activated myofibroblasts are identified as the predominant effector cells and prompt the deposition of extracellular matrix (ECM).^1^ However, current myofibroblast‐centered views for antifibrotic therapy are not sufficient for the treatment of the majority of patients with fibrosis. Interestingly, macrophage heterogeneity is commonly observed in the pathogenesis of fibrotic diseases and can either attenuate or exacerbate fibrosis progression.

Monocyte/macrophage plays key roles in innate immune system and is characterized by phenotypic diversity and functional plasticity. There are two principal macrophage subsets with opposite activation states are known as classical (M1) and alternative (M2) phenotypes.^2^ M1/M2 polarization represents the extremes of a continuum of functional states in response to different microenvironmental signals. M1 subset is stimulated by microbial products or pro‐inflammatory cytokines, such as interferon‐γ (IFN‐γ), tumor necrosis factor α (TNF‐α), or toll‐like receptor (TLR) ligands, thereby suggesting a role of proinflammation and resistance against intracellular parasites and tumors.^3^ By contrast, macrophages exposed to interleukin (IL)‐4, IL‐10, IL‐13 or transforming growth factor (TGF)‐β differentiate toward an M2 phenotype. The outcome of an M2 polarizing event is tightly linked to anti‐inflammatory response, tumor progression, tissue repair, and remodeling.^4^ M1 or M2 phenotype is not fixed and can be reversed in the context of specific stimuli. For example, gene expression analysis confirmed that macrophages could undergo M1 to M2 transition after removing the inflammatory cues in the local microenvironment.^5^ Heterogeneous macrophages are commonly involved in the pathogenesis of different fibrotic diseases and can function as either promoter or suppressor of fibrosis across different organ types.

Noncoding RNAs (ncRNAs) represent an important population of the transcriptome, which are comprised of a wide range of endogenous RNA‐based molecules. Different ncRNAs are emerging as a revolution in the regulation of gene expression and are involved with M1/M2 polarization.^6^ Here, we summarize the phenotype and ontogeny of different macrophage subpopulations and discuss the key roles and molecular mechanisms of ncRNAs action in M1/M2 polarization in the context of fibrotic microenvironment including liver, kidney, lung, and heart. Characterization of ncRNA‐mediated macrophage heterogeneity may contribute to developing novel opportunities for their therapeutic translation for fibrotic diseases.

## 2 THE CLASSIFICATION AND FUNCTION OF NCRNAS

**Figure 1:**
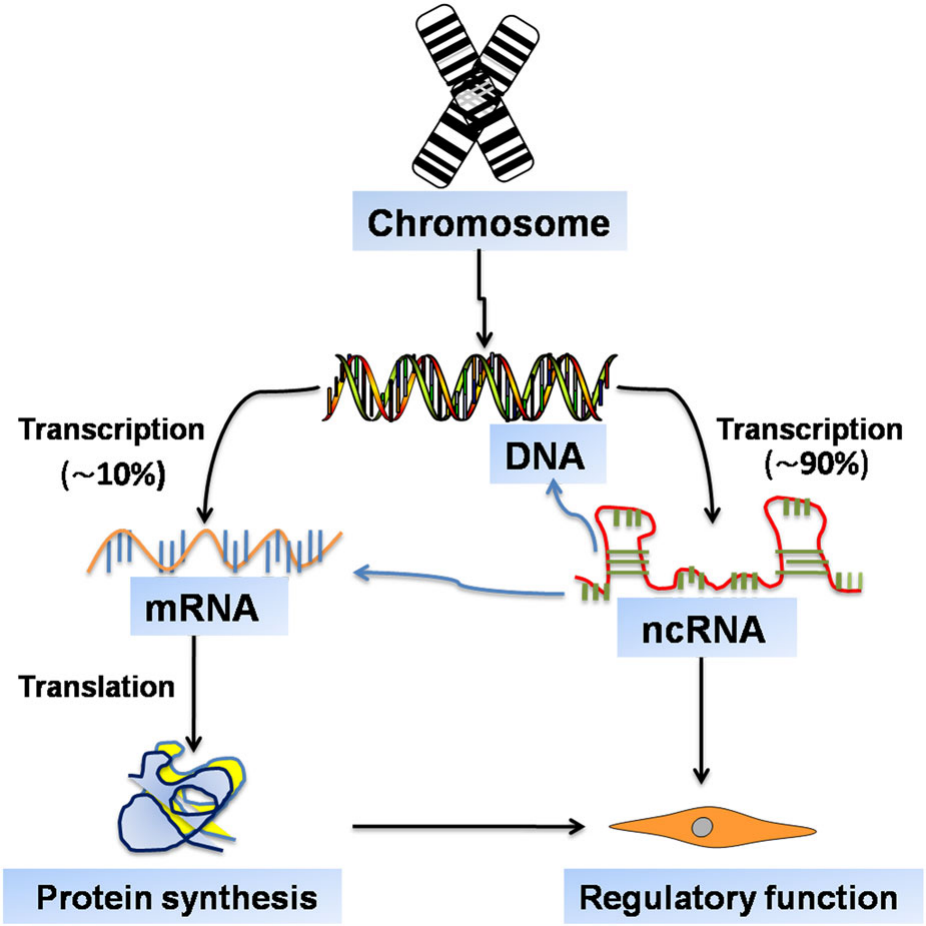
Noncoding genes account for most transcription from the genome. Eukaryotic genomes are extensively transcribed, forming both messenger RNAs (mRNAs) and noncoding RNAs (ncRNAs). Of note, ncRNAs remarkably differ from their better‐known counterpart mRNAs, including transcripts numbers and functions. Although these ncRNAs that do not code for proteins, they may affect gene expression and disease progression through a variety of mechanisms

Noncoding portion of the mammalian genome, rather than its coding counterpart, is likely to explain the greater complexity of higher eukaryotes. Among all epigenetic modifications, ncRNAs are undeniably one of the best‐studied mediators of innate immune system, which are not protein‐coding genes (PCGs) and accounted for almost 90% across the human transcriptome.^7^ To date, a growing number of ncRNAs is known to participate in the control of cell biology, including long noncoding RNA (lncRNA), microRNA (miRNA), and circular RNA (circRNA). Pervasive expression of different types of ncRNAs is a prominent feature of the gene regulatory networks of multicellular organisms. Given the critical role of ncRNAs in regulating gene expression, harnessing these regulatory responses promotes the dissected research field of ncRNA‐targeted therapy potency (Figure 1).

**Figure 2:**
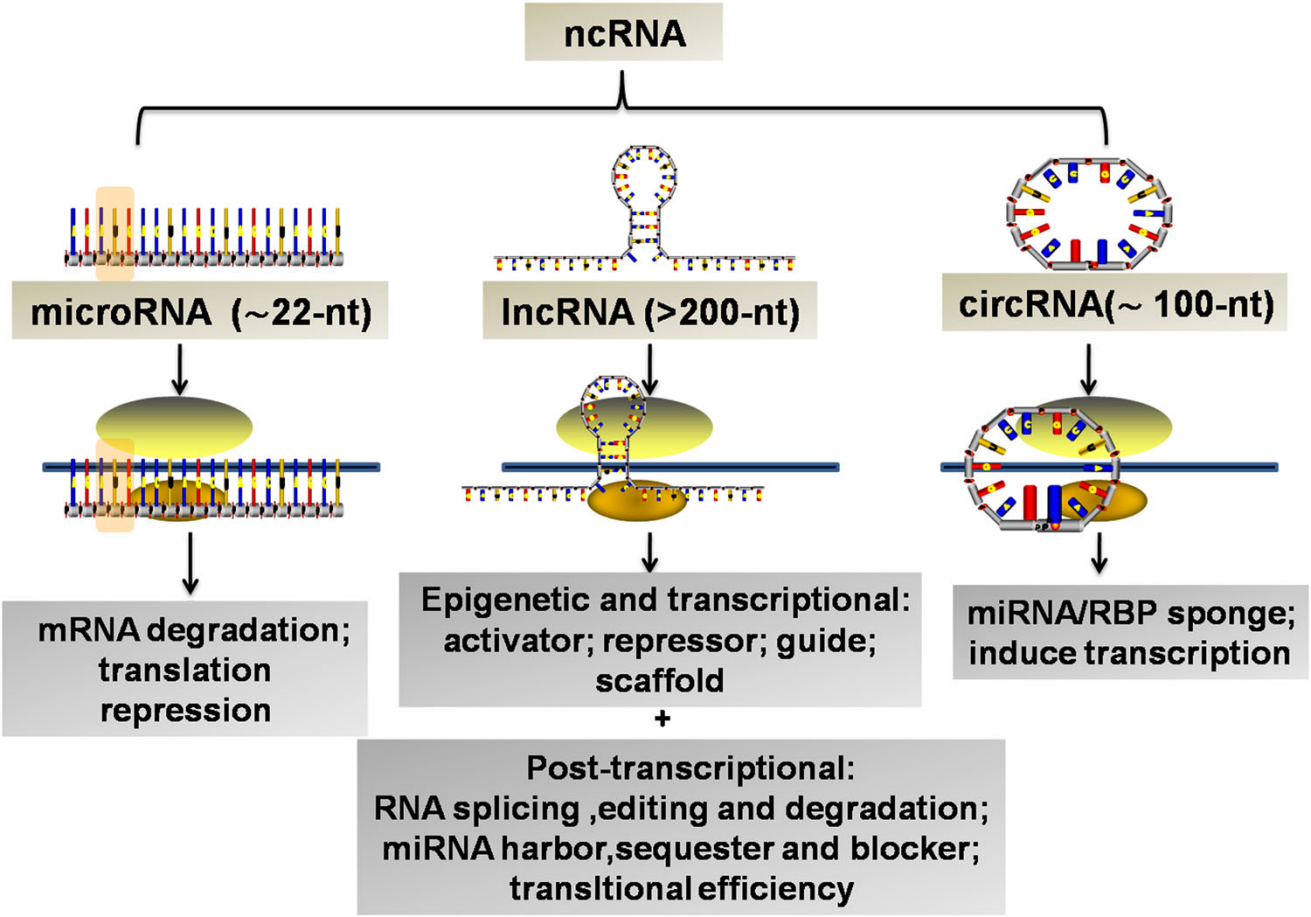
An expanding universe of ncRNA classification and function. Accumulating evidence has uncovered the presence and importance of ncRNAs, which includes miRNAs, lncRNAs, and circRNAs. Intensive research studies have revealed that different ncRNAs play key roles in a great variety of processes, including transcriptional regulation, chromosome replication, RNA processing and modification, mRNA stability and translation, and even protein degradation and translocation. circRNA, circular RNA; lncRNA, long noncoding RNA; mRNA, messenger RNA; miRNA, microRNA; ncRNA, noncoding RNA; RBP, RNA‐binding protein

Firstly, miRNAs are small ncRNAs molecules of ∼22 nucleotides in length and are evolutionarily conserved across species.^8^ They negatively regulate gene expression by sequence‐specific translation inhibition and mRNAs decay by binding 3′ untranslated regions (UTRs). In addition, lncRNAs have emerged as key components of ncRNAs and play a critical function in the gene activation and deactivation. They are generated by RNA polymerase‐mediated extragenic transcription and at least ∼200 nt in size.^9^ LncRNAs could cause the cis action on the genome and chromatin, which are capable of regulating several biological phenomena, such as gene imprinting and transcriptional enhancement by acting as molecular scaffolds, architectural RNAs, or as regulatory molecules.^10^ In particular, they have the ability to compete for miRNA binding by acting as a competing endogenous RNA (ceRNA) and “sponges” for miRNAs.^11^ More recently, circRNAs have been attracting much interest for their potential in the maintenance of diseases and homeostasis. They are produced by circularization of specific exons of 3′ and 5′ ends covalently bonded and are highly abundant and evolutionarily conserved. However, the role of circRNAs in gene regulation is still not completely understood and some research studies imply their functions in acting as a miRNA sponge and regulating RNA‐binding proteins.^12^ The coordinated activities of ncRNA‐mediated M1/M2 polarization are essential for the maintenance of tissue homeostasis and are also associated with the development of inflammatory and fibrotic disorders.^13^ There is an urgent need to improve our understanding of the biological function of more potent ncRNAs. Herein, we review different ncRNAs molecules that are capable of regulating macrophage polarization in a variety of fibrotic diseases. These investigations into the mechanisms of how these ncRNAs determine specific macrophage phenotypes, hold promise for the treatment of fibrosis across different organ types (Figure 2).

## 3 HEPATIC FIBROSIS

Hepatic fibrosis (HF) is the common outcome of various liver injuries, and might progress to cirrhosis and liver cancer. Liver parenchymal cells (ie, mainly hepatocytes) and nonparenchymal cells (ie, mainly hepatic stellate cells [HSCs] and various immune cells) are totally responsible for maintaining liver homeostasis and diseases. Activated HSCs have been identified as the most important promoter in the process of liver fibrogenesis by releasing abundant ECM.^14^ HF is widely regarded as a reversible wound‐healing response by selectively inducing HSCs apoptosis, whereas incomplete clinical effects are obtained. Fine‐tuning of the balance between two functionally contrasted hepatic macrophage subsets is now at the heart of macrophage‐based antifibrotic therapy. Infiltrating monocyte‐derived macrophages and resident Kupffer cells (KCs) have been implicated in the pathogenesis of liver inflammation and fibrosis, either by promoting inflammatory pathways with M1 subset, or by enhancing anti‐inflammatory response with M2 subset. Furthermore, the ncRNA‐dependent M1/M2 polarization is required for causing either profibrotic or antifibrotic responses in HF microenvironment.^15^

High levels of sphingosine kinase (SphK1) promotes the activation and migration of HSCs and KCs by inhibiting miR‐19b‐3p, resulting in the enhanced secretion of CCL2 and CCR2.^16^ CCL2/CCR2 axis is frequently found to cause the blood monocytes' recruitment into inflamed tissues and promotes M2 polarization. Indeed, the CCR2+ macrophage pharmacologic antagonist exhibits a significant antitumor function for hepatocellular carcinoma (HCC).^17^ The detection of miRNAs is a prime example of the use of macrophage activation pathways to drive the recognition of the pathophysiology of alcoholic liver diseases (ALDs). MiR‐155 is a gene inducible by many stimuli such as TLR4 and links alcohol‐induced responsiveness and inflammation of KCs. As expected, upregulation of miR‐155 in KCs of the chronic alcohol‐exposed livers contributes to the elevation of TNF‐α by nuclear factor kappa B (NF‐κB) activation and targeting C/EBPβ in ALD.^18^ Conversely, miR‐155 deficiency significantly inhibits the alcohol or methionine‐choline–deficient (MCD) induced steatohepatitis and fibrosis by decreasing the number of CD163+ CD206+ infiltrating macrophages and promoting M2 KCs.^19^ In addition, a predominant M2 KC profile existed in the miR‐155‐deficient mice, which could ameliorate liver ischemia‐reperfusion (IR) injury by suppressing pro‐inflammatory cytokine (TNF‐α, IL‐6, and IL‐1β) secretion and enhancing IL‐10 production.^20^ These observed results seem to uncover the potential therapeutic role of miR‐155 in HF microenvironment. Another human study showed that acute alcohol binge induced the significantly increased expression of miR‐27a in monocytes, which appeared to involve attenuated M1 and enhanced M2 polarization by targeting sprouty2 and ERK pathway activation.^21^ Hepatic schistosomiasis is hallmarked by the hepatic granulomas and fibrosis, which could be prevented by the elevated miR‐146a/b‐dependent M2 KC polarization through targeting signal transducer and activator of transcription 1 (STAT1).^22^ The influence of the different miRNAs on the hepatic macrophage functional plasticity has been proposed as a promising landscape for disrupting liver inflammation and HF.

Consistent with the above findings, the latter cases may also demonstrate that hepatic macrophages could undergo different polarization following the changes of miRNA signaling and function in other disease conditions. Relevant to this, the miR‐15a/16^−/−^ mice have been observed to exhibit retarded transplanted hepatic cancer (H22) cells growth and increased sensibility to dextran sulfate sodium (DSS)‐induced colitis, resulting from the M1 polarization by the coactivation of NF‐κB and STAT3.^23^ NF‐κB activation has also proved effective in enhancing M1 polarization in obstructive sleep apnea (OSA), and exacerbates inflammation and fibrosis in patients with nonalcoholic steatohepatitis (NASH) by inhibiting miR‐365.^24^ It is therefore that inflammatory M1 polarization might display antitumor activities. For example, a study in mice has shown that elevation of liver macrophage miR‐138 exacerbates acute liver failure (ALF) by suppressing p53 and enhancing inflammatory factors (TNF‐α, IL‐6, and IL‐1β) expression.^25^ Furthermore, overexpression of MCP‐1 induced protein (MCPIP1) in KCs could alleviate the lipopolysaccharide (LPS)‐induced live injury and septic mice by negatively regulating miR‐9/SIRT1 pathway.^26^ The serum and peripheral blood monocytes (PBMs) of patients with chronic hepatitis B (CHB) had decreased miR‐210 levels, which could inhibit the anti‐inflammatory macrophage activation.^27^ Silencing of miR‐375 could decrease the apoptosis of KCs and the IL‐6, TNF‐α, and IL‐1β expressions by targeting astrocyte elevated gene‐1 (AEG‐1), which improves immune function in mice with ALF.^28^ Adipose mesenchymal stem cell (AMSC)‐secreted exosomes (AMSC‐Exo) contained high levels of miR‐17 which could reduce ALF through suppressing nucleotide‐binding and oligomerization domain‐like receptor 3 (NLRP3) inflammasome activation in KCs by the inhibition of TXNIP.^29^

Apart from miRNAs, other ncRNAs (including lncRNAs, cirRNAs) have been considered to be a putative strategy to affect the function of hepatic macrophages. Mice lacking the lncRNA AK139328 show reduced liver IR injury by the molecular events including decreased macrophage infiltration, inhibited NF‐κB activity and inflammatory cytokines expression.^30^ Extracellular vesicles (EVs) can mediate the transfer of some lncRNAs, which is capable of cell‐to‐cell communication in liver disease. HCC cell‐derived exosomes contain elevated levels of lncRNA TUC339, which leads to the decreased pro‐inflammatory cytokine production and enhanced M2 polarization.^31^ Microarray analysis has identified that lncRNA TUC339 promotes M2 activation caused by the decreased phagocytosis, involving TLR signaling, cytokine‐cytokine receptor interaction, chemokines and their receptor signaling pathway.^31^ LncRNA COX‐2 upregulation is positively correlated with the progression of fibrotic area. LncRNA COX‐2 is known to exert profibrotic function in HF and M1 polarization mechanism might be required in this process. Indeed, Ye et al,^32^ found that M1 macrophages coincubation with HCC cell could inhibit the HCC proliferation, invasion, migration, epithelial‐mesenchymal transition (EMT) dependent on the upregulated lncRNA COX‐2. Transcribed ultraconserved regions (T‐UCRs) uc.306 is a subset of lncRNAs and its deficiency is significantly associated with a shorter overall survival of HCC due to the M2 polarization.^33^ These findings have prompted studies directed toward the identification of the specific patterns of lncRNA expression in mediating M1/M2 KC polarization in different liver diseases. So far, circRNAs have been suggested to represent specific modulator of macrophage inflammation in HF, such as mmu‐circ‐35216, ‐42398, ‐34116, and ‐30981.^34^ In the LPS‐induced inflammatory injury model in RAW264.7 cells, one circRNA (mmu‐circ‐35216) expression is significantly increased, three circRNA (mmu‐circ‐42398, ‐34116, and ‐30981) expression is significantly decreased. These differentially expressed circRNAs are involved in the cell composition, biological processes, molecular function, and several cell signal pathways.^34^

**Figure 3:**
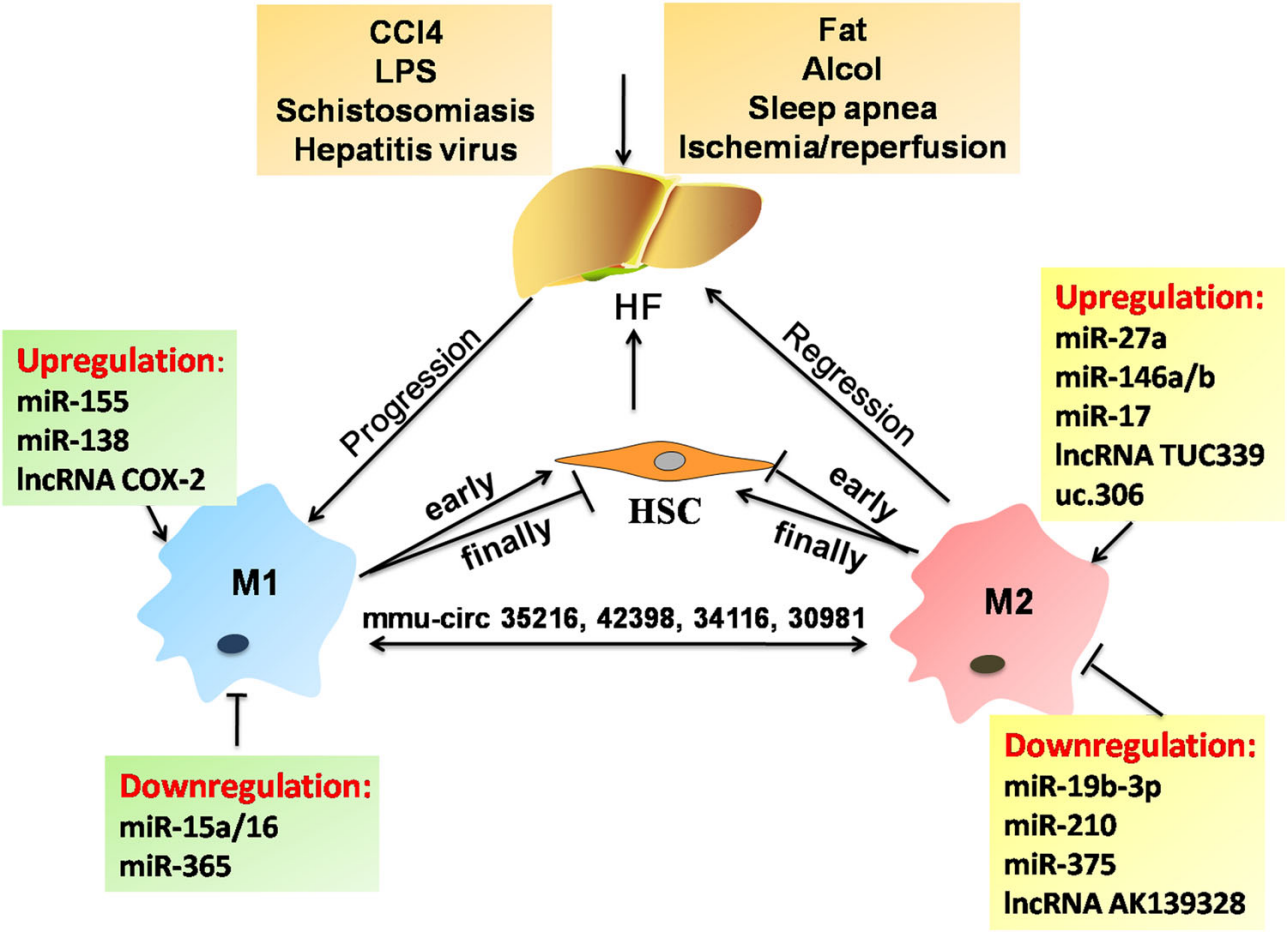
Crosstalk between heterogeneous macrophages and ncRNAs in liver fibrosis. Liver fibrosis could be induced by different etiologies (CCl4, LPS, schistosomiasis, hepatitis virus, etc). Heterogeneous macrophages (M1 and M2) contribute to the progression and regression of liver fibrosis by regulating the proliferation and activation of HSCs. HF, hepatic fibrosis; HSC, hepatic stellate cell; LPS, lipopolysaccharide; ncRNA, noncoding RNA

A novel ligand of CCR2, PC3‐secreted microprotein/microseminoprotein (PSMP/MSMP), which can give rise to inflammatory macrophage infiltration and pro‐inflammatory cytokines production in HF patients.^35^ The antibody of PSMP is a potential therapeutic agent for the treatment of liver fibrosis. In nonalcoholic fatty liver disease patients, sialic acid‐binding immunoglobulin‐like lectin‐7 (Siglec‐7) was mainly expressed on CCR2+ macrophages in the liver and serum levels of soluble Siglec‐7 (sSiglec‐7) were increased after stimulation by pro‐inflammatory factors in macrophages, which could serve as an independent and therapeutic marker with high specificity for advanced HF in this patient population.^36^ To date, most of the data are obtained from murine models, and studies employing human patients are still not enough. Further studies will be necessary to fully understand the critical roles of various ncRNA‐mediated macrophage polarization for the identification of new regulatory networks in HF and disease progression (Figure 3).

## 4 PULMONARY FIBROSIS

Pulmonary fibrosis (PF) is a chronic and highly heterogeneous respiratory disease characterized by abnormal wound‐healing condition with high mortality rates. The prevailing view has been that lung myofibroblasts are a major contributor to the aberrant deposition of ECM in PF. This long‐held view of antifibrotic therapy by directly targeting lung myofibroblasts has been challenged by evidence for a dual role of alveolar macrophages (AMs) in the pathophysiology of PF, either pro‐inflammatory or anti‐inflammatory effects.^37^

Fibrotic changes in the lungs are developed from exposure to various conditions (irradiation, toxin, silica, cigarette) and are associated with M2 polarization. TGF‐β1 is extensively involved in the development of PF, stimulating ECM synthesis through a series of intracellular signaling molecules. TGF‐β1 has been implicated in mediating the differentiation and homeostasis of AMs and macrophage‐derived TGF‐β1 promotes PF.^38^ Evidence suggests that bleomycin (BLM)‐induced PF in miR‐155^−/−^ mice develop exacerbated PF, which is contributed by the liver X receptor (LXR)α deregulation and TGF‐β1 production in AMs.^39^ The diverse functions of miR‐155 in different tissues fibrosis by targeting macrophage activation, remain to be explored. Next, miR‐140 has been found to be downregulated in radiation‐induced lung fibrosis (RILF), resulting in the M2 polarization and RILF progression by activating TGF‐β1/Smad3.^40^ These results suggest that miRNA‐mediated TGF‐β1 activation is required for induction of AMs polarization in PF.

Recent data demonstrate that alcohol increases susceptibility to lung infection through the enhanced levels of miR‐130a/‐301a, and is capable of promoting the upregulation of TGF‐β1 by targeting peroxisome proliferator‐activated receptor (PPAR)‐γ in AMs.^41^ PPARγ activation is essential for miR‐27‐3p‐mediated TLR‐2/4 signaling cascades and involves the M1‐like AMs activation and pulmonary inflammation.^42^ TLR signaling molecules have been deemed as potential targets involving the fine‐tuning lung inflammatory response regulated by multiple miRNAs in AMs. Study in LPS/cigarette smoke (CS)‐treated acute lung injury (ALI) rats have shown that AMs display significant upregulation of miR‐21 and miR‐344b‐1‐3p, which could inhibit the inflammatory responses by targeting TLR‐2/4 and NF‐κB signaling pathway.^43^ Microarray mRNA results indicated that AMs of smokers and chronic obstructive pulmonary disease (COPD) patients are defined by decreased M1‐ and increased M2‐regulated transcripts, along with the reduction of global miRNAs.^44^ This observation agrees well with the fact that circulating miR‐320a secreted by neutrophils of smokers can modify the macrophages to an M2‐like protumorigenic phenotype through downregulation of STAT4.^45^ Celecoxib ameliorated lung hyperinflammation in cystic fibrosis patients, which was caused by decreasing miR‐199a‐5p levels in the PI3K/AKT‐dependent manner in CF macrophages.^46^ Furthermore, upregulated miR‐203 can alleviate lung injury in septic shock mouse models by activating AKT signaling pathway and reducing AMs levels.^47^ Deletion of miR‐127 was evident in impairing M1 and enhancing M2‐biased phenotype, resulting in a decreased pulmonary inflammation and injury by the activation of JNK activity.^48^ The key role of different miRNAs is well established in defining AM polarization and dissection of the molecular mechanisms may pave the way to translation. For instance, Yao et al^49^ discovered the ability of exosomes to transfer overexpressing miR‐328 from M2 AMs to pulmonary interstitial fibroblasts, thereby triggering fibroblast proliferation and aggravating PF through the regulation of FAM13A. Silicosis is pathologically characterized by the diffused PF and silica‐treated macrophages induce fibroblast activation through the expression of MyD88 and Smad3 by inhibiting miR‐29b and miR‐489.^50^, ^51^ Distinct lncRNA signatures are associated with macrophage inflammatory response in LPS‐induced ALI, suggesting that lncRNAs might also alter the AM phenotypes. It has recently been shown that lncRNA MALAT1 could ameliorate BLM‐induced PF by suppressing M2 AMs and profibrotic genes.^52^ Mechanistically, MALAT1 knockdown promotes IL‐4 induction of mitochondrial pyruvate carriers and their mediation of glucose‐derived oxidative phosphorylation (OxPhos) is critical for MALAT1‐regulated M2 polarization.^52^ In consistent, MALAT1 has been known to elicit M1 activation and exacerbate the septic lung injury in mice. MALAT1 functioned as a molecular sponge for miR‐146a and activated the p38 MAPK/p65 NF‐κB signaling pathway.^53^ Therefore, MALAT1‐associated M1 polarization is involved in different pulmonary pathogeneses and play opposite roles in pulmonary injury and fibrosis. There is a negative feedback loop underlying the transcript isoforms of lncRNA MEG3, transcript 4 (MEG3‐4)‐mediated inflammatory cytokines production by the sponging of miR‐138 in macrophages, which could prevent sepsis following lung infection.^54^ MEG3‐4‐mediated decoy and sponging of miR‐138 in the cytoplasm increases the IL‐1β expression that subsequently induces a negative feedback mechanism mediated by NF‐κB that decreases MEG3‐4 abundance and inflammatory cytokine production.^54^ M2‐derived TGF‐β1 could stimulate the upregulation of lncRNA‐ATB in lung epithelial cells and the latter exacerbated PF by promoting the EMT and targeting miR‐200c/ZEB1 axis.^55^ LncRNA GNAS‐AS1 is crucial for non–small‐cell lung cancer (NSCLC) progression by directly inhibiting miR‐4319, which could target N‐terminal EF‐hand calcium‐binding protein 3 (NECAB3) to inhibit its expression and induce the tumor‐promoting M2 polarization.^56^

**Figure 4:**
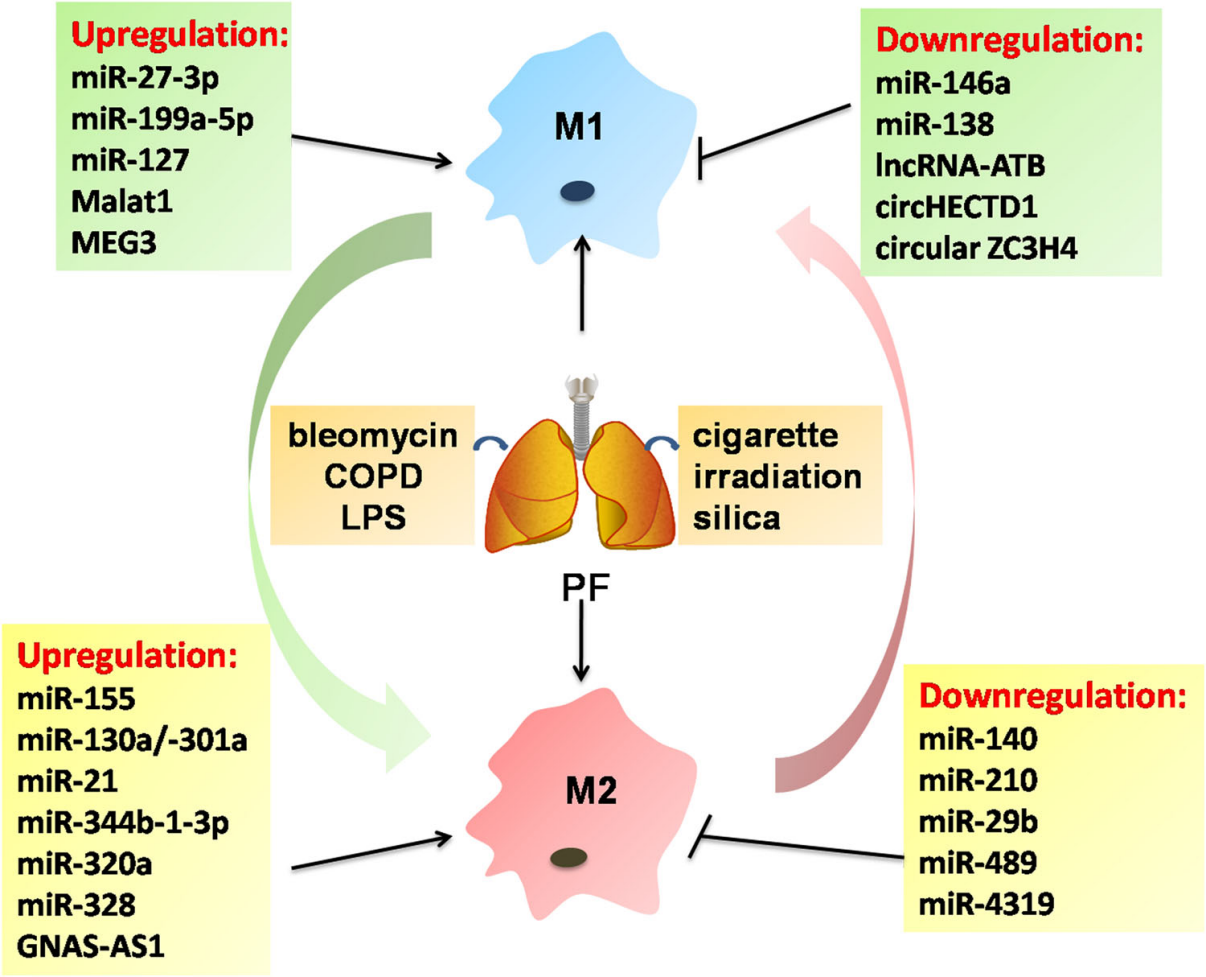
Emerging roles for ncRNAs in pulmonary fibrosis by targeting heterogeneous macrophages. Pulmonary fibrosis is now generally regarded as a consequence of multiple risk factors, such as cigarette smoke, irradiation, and silica. ncRNAs have been implicated in the pathogenesis of pulmonary fibrosis, which is associated with the function of heterogeneous macrophages. COPD, chronic obstructive pulmonary disease; lncRNA, long noncoding RNA; LPS, lipopolysaccharide; miR, microRNA; ncRNA, noncoding RNA

Differentially expressed circRNAs have further amplified the unique ncRNAs functions in shaping AMs activation under physiological and pathological conditions. SiO_2_‐induced macrophage activation is capable of promoting fibroblast proliferation and migration via the circHECTD1/HECTD1 pathway ubiquitination^57^ and circular ZC3H4 RNA/miR‐212/ZC3H4 pathway.^58^ M2‐like macrophage markers (CD163 and CD204) and CD163/CD68 and CD204/CD68 cell ratios are significantly elevated in idiopathic pulmonary fibrosis (IPF) patients, associating with shorter overall survival and time‐to‐acute exacerbation in IPF patients.^59^ Macrophages have a dual action in mounting a pro‐inflammatory M1‐like response to lung injury as well as in the repair of injury and profibrotic M2‐like effects in the lung. Given the crucial role of macrophage polarization in the development of PF, harnessing the ncRNA‐mediated M1/M2 responses opens up new possibilities for PF control (Figure 4).

## 5 RENAL FIBROSIS

Renal fibrosis (RF) has been implicated in different chronic kidney diseases (CKDs) and is characterized by excessive ECM deposition within the glomerulus and interstitium. Activated myofibroblasts is a key driver of ECM components in RF, of which a large part is due to the complex fibroblast‐macrophage transdifferentiation and interaction. For example, TGF‐β/Smad signaling mediates the transition of bone marrow‐derived M2‐type macrophages to myofibroblasts in the renal allograft.^60^ Emerging evidence suggests that M1/M2 renal macrophages (RMs) and infiltrated macrophages (IMs) are necessary in regulating kidney inflammation and fibrosis. Bone marrow‐derived mesenchymal stem cells (BM‐MSCs) could ameliorate IR injury in kidney through the induction of M2 polarization.^61^ Resident adult renal/progenitor cells (ARPCs) have been recently identified as a promising population in preventing endothelial‐to‐mesenchymal transition process and promoting kidney repair in both sepsis‐ and endotoxemia‐induced acute kidney injury (AKI).^62^ However, the immunomodulatory effect of ARPCs on macrophages has been still largely unknown. Interestingly, miRNAs are increasingly deemed as potential mediators in the kidney macrophage activation and function. Downregulation of miR‐376b/Atg5 suppresses renal interstitial fibrosis (RIF) by promoting RM autophagy.^63^ Obesity‐induced nephropathy could be inhibited by suppressing miR‐802 or miR‐155 through NF‐κB signaling, which is associated with the reduction of IMs.^64^, ^65^ Silencing of miR‐21a‐5p/Notch2 receptor and overexpression of miR‐374a may be viable therapeutic options in the treatment of chronic renal allograft dysfunction and diabetic nephropathy (DN), as indicated by a reduction in IM influx.^66^ Human umbilical cord‐derived MSCs attenuated RF occurring in AKI associated with reduced macrophage infiltration by downregulating miR‐29a and miR‐34a.^67^ In summary, the above studies suggest that exploration of the full spectrum of miRNAs in macrophage polarization has served as a paradigm of macrophage plasticity and RF.

Under many conditions, TGF‐β1 is a critical mediator of RF and correlates with the aberrant expression of miRNAs. For example, overexpression of miR‐146a in splenic macrophage significantly inhibits the sepsis‐related renal injury.^68^ Exosomes released from high glucose (HG)‐stimulated macrophages are responsible for the activation of glomerular mesangial cells and DN progression through TGF‐β1/Smad3 pathway.^69^ Intriguingly, tubular epithelial cell (TEC)‐derived miRNA‐23a and miR‐19b‐3p–containing exosomes both lead to M1 macrophage activation and tubulointerstitial inflammation by targeting ubiquitin editor A20^70^ and SOCS1,^71^ respectively. M2‐derived legumain ameliorates the deposition of collagen and fibronectin induced by ureteral obstruction (UO) and subsequently mediates the antifibrotic effect of M2 macrophages.^72^ IL‐1 receptor‐associated kinase (IRAK)‐M–deficient mice are protected from RF that is associated with decreased M2 polarization in UO.^73^ MiR‐146a KO mice in a model of streptozotocin (STZ)‐induced diabetes has displayed exacerbated RF than wild‐type mice, resulting from the suppression of M1 genes (IL‐1β, IL‐18) and increased expression of M2 markers.^74^ Pioglitazone could decrease renal calcium oxalate crystal formation and renal inflammation by reducing IMs and M1 RM polarization in the kidney, through a PPAR‐γ‐miR‐23‐interferon regulatory factor 1/Pknox1 axis.^75^ Eventually, tumor‐associated macrophages (TAMs) plays a key role in carcinogenesis of renal cell carcinoma by inhibiting miR‐486‐5p levels in kidney cancer cells through the induction of CCL2.^76^

**Figure 5:**
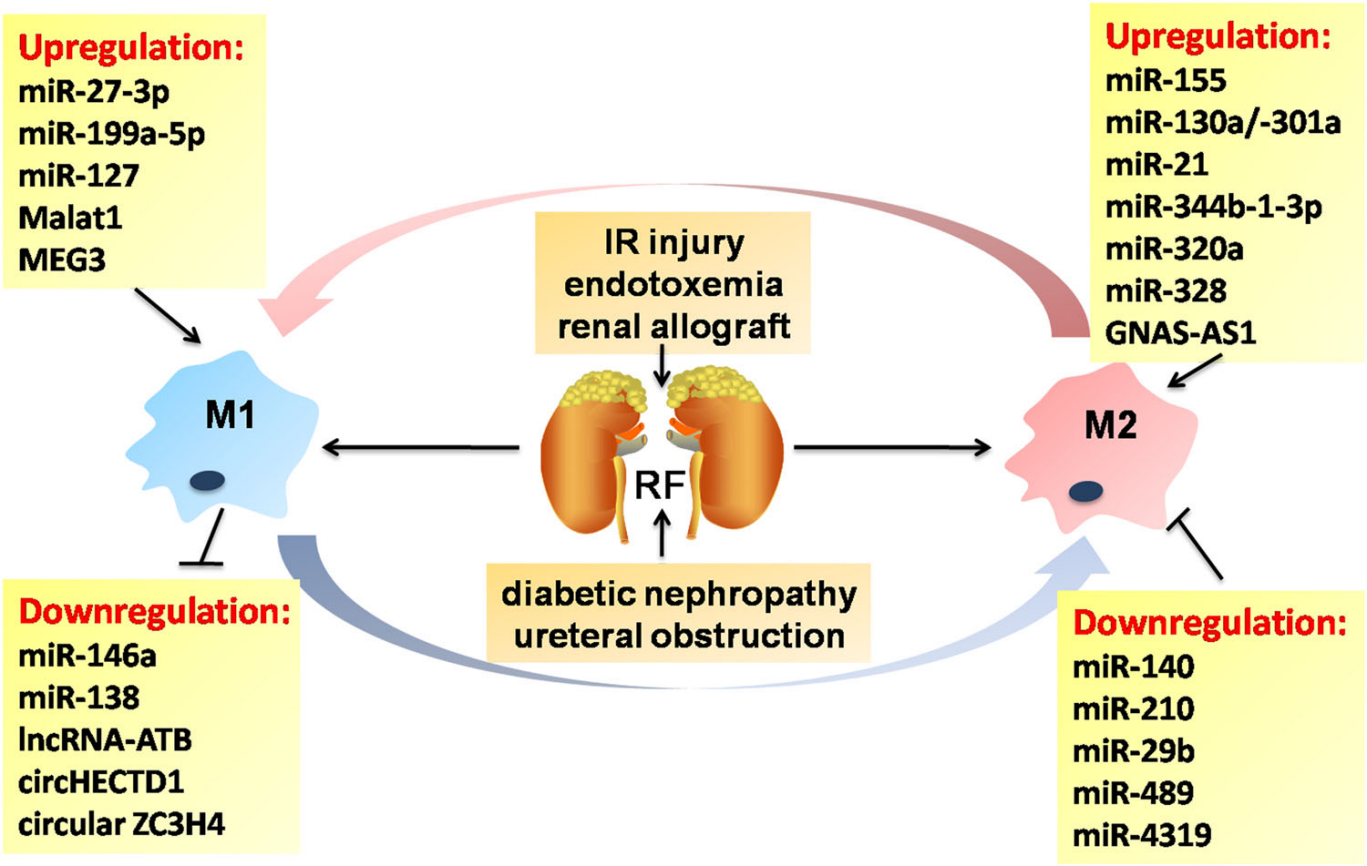
Noncoding RNA‐mediated macrophage phenotypic regulation in renal fibrosis. Renal fibrosis contributes greatly to end‐stage renal failure, characterized by the excessive ECM deposition in the interstitium of kidney. In response to the different injuries, infiltrating and resident macrophages could undergo M1 or M2 polarization, which is largely dependent on the regulation of multiple noncoding RNAs. ECM, extracellular matrix; IR, ischemia‐reperfusion; RF, renal fibrosis

Many lncRNAs are garnering increasing attention for their dysregulated expression in the pathogenesis of RF and disease progression, which could exert either pro‐inflammatory or profibrotic effects. Notably, the upregulation of lncRNA E330013P06 was found in monocytes from type‐2 diabetes patients and mouse macrophages treated with HG and palmitic acid.^77^ It promoted a dysfunctional M2 phenotype (decreased IL‐10 levels) and enhanced M1 inflammatory response (increased IL‐6, TNF, PTGS2, and CCL2 levels) in macrophages, which could develop lncRNA‐based therapies for inflammatory diabetic complication.^77^ LncRNA LRNA9884 and Erbb4‐IR are both Smad3‐dependent lncRNAs that promoted renal inflammation and fibrosis in DN by triggering MCP‐1 production and suppressing miR‐29.^78^, ^79^ Overexpression of lncRNA NR_038323 ameliorates the RF in STZ‐induced DN via miR‐324‐3p/DUSP1/p38MAPK and ERK1/2 pathway.^80^ LncRNA Mirt2 functions as a checkpoint to prevent aberrant activation of inflammation and inhibit endotoxemia‐induced fatality and multiorgan dysfunction including kidney and liver.^15^ The treatment of obstructive kidneys in mice with quercetin, decreases the levels of iNOS and IL‐12, as well as the proportion of F4/80+/CD11b+/CD86+ macrophages by downregulating NF‐κB and thereby inhibits the M1 polarization.^81^ Moreover, quercetin also inhibits the polarization of F4/80+/CD11b+/CD206+ M2 macrophages by antagonizing the TGF‐β1/Smad2/3 signaling, which may have therapeutic potential for patients with kidney injury and fibrosis.^81^ Thus, future clinical studies will need to address whether the above ncRNA‐based approaches to promote the M1/M2 polarization in humans may generate new therapeutic strategies for RF (Figure 5).

## 6 CARDIAC FIBROSIS

Cardiac fibrosis (CF) is central to various heart diseases and is characterized by a net accumulation of ECM in the cardiac interstitium. Consistent with the role of myofibroblasts in other tissues, cardiac myofibroblasts are the predominant ECM‐producing effector cells and are responsible for the development of CF. The emerging role of infiltrating macrophages and resident cardiac macrophages (CM) in the activation of fibroblasts, suggests that distinct macrophage lineages represent promising targets for cardiac injury, recovery, and remodeling.

Trimethylamine *N*‐oxide (TMAO), a gut microbe‐derived metabolite, leads to the deteriorated CF through accelerating the transformation of fibroblasts into myofibroblasts and macrophage activation by targeting TGF‐β/Smad pathway.^82^ TGF‐β/Smad3 activation in macrophages protects the infarcted heart from adverse remodeling by promoting an anti‐inflammatory M2 phenotype.^83^ Differentiation of M1/M2 macrophages in the myocardium has been associated with the development of CF and the underlying mechanisms have also been a topic of intensive research. There is evidence that miRNAs regulate macrophages polarization and infiltration (miR‐21,^84^ miR‐133a^85^) and is involved in CF. Firstly, inhibition of miR‐155 decreases myocardial infarction (MI)‐induced sympathetic neural remodeling by repressing M1 polarization‐dependent on the SOCS1/NF‐κB pathway.^86^ Hearts of microRNA‐155(−/−) mice are shown to the decreased susceptibility to viral myocarditis and improved cardiac function by modulating M2 polarization.^87^ Tail vein injection of miR‐155 inhibitor, miR‐155‐AuNP, could reduce cell apoptosis, CF, and restore the cardiac function by enhancing M2 ratio in ovariectomized diabetic mice.^88^ Local delivery of a miR‐21 mimic using nanoparticle or ultrasound‐targeted microbubbles into lesion sites attenuates post‐MI remodeling, heart failure, and atherosclerosis by switching macrophage phenotype from pro‐inflammatory M1 to reparative M2.^89^

**Figure 6:**
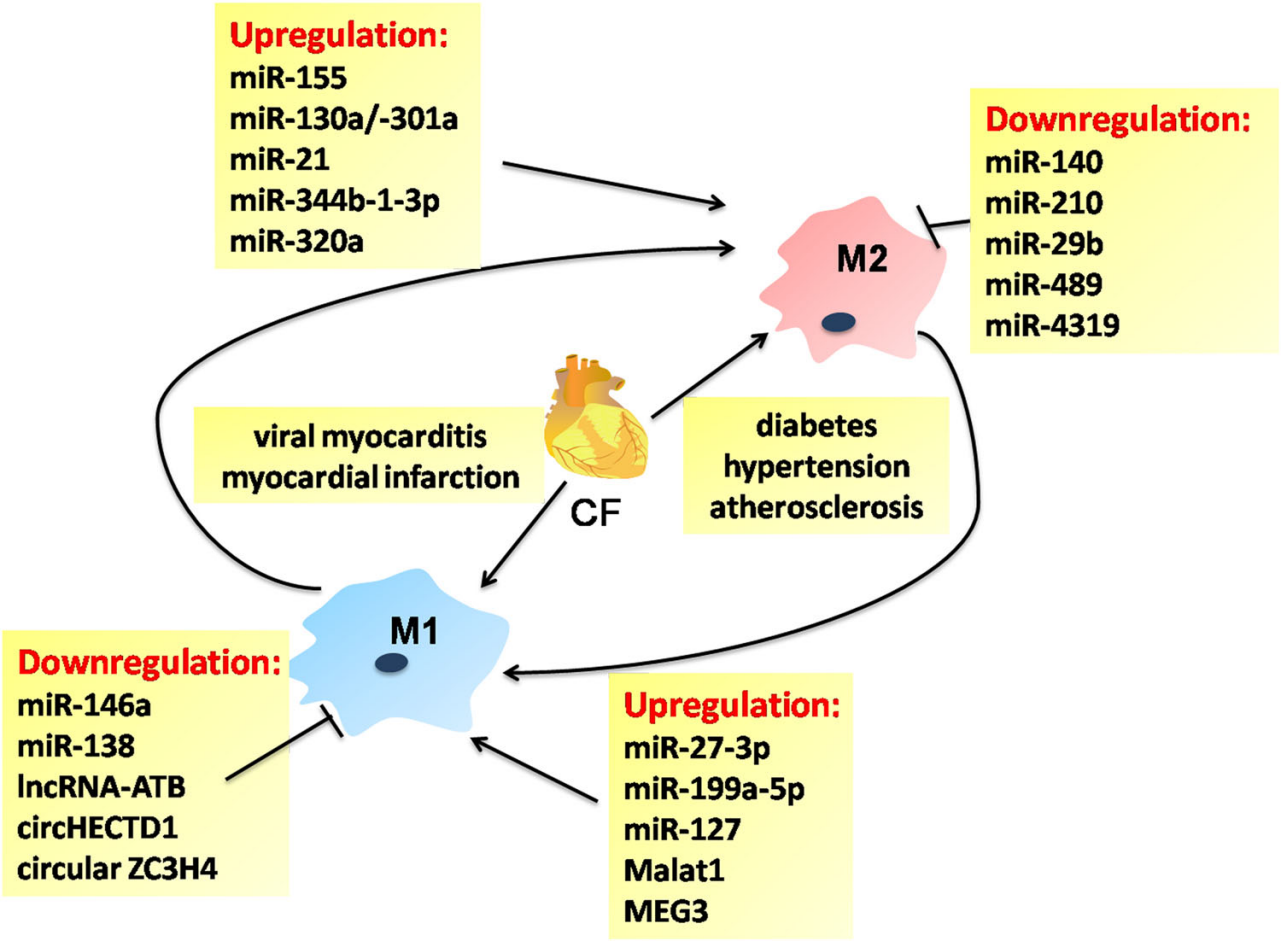
Noncoding RNA‐associated macrophage polarization regulatory networks in cardiac fibrosis. Cardiac fibrosis (CF) is considered as an important and early risk factor for cardiac development and diseases, triggered by variable etiology. Noncoding RNAs play an important role in the pathogenesis of CF by targeting M1 or M2 polarization

In addition, lncRNAs have attracted great interest as biomarkers and targets for preventing cardiac remodeling and fibrosis by modulating macrophage inflammatory functions.^90^ For instance, levels of lncRNA H19 in peripheral blood mononuclear cells (PBMCs) are elevated in the coronary artery disease (CAD) patients and considered as potential biomarker for CAD diagnosis and prognosis.^91^ Sallam et al^92^ indicated that loss of lncRNA MeXis in mouse bone marrow cells damaged LXR‐dependent genes transcription and accelerated the development of atherosclerosis. Interestingly, lncRNA MALAT1 and NEAT1 have been found to serve as novel immunoregulators affecting monocyte‐macrophage functions and their disruption may contribute to identifying high risk in post‐MI and atherosclerosis patients.^93^, ^94^ In the treatment of LPS, NEAT1−/− bone marrow‐derived macrophages (BMDMs) displayed increased reactive oxygen species production and disturbed phagocytic activity following altered transcriptomes, along with aberrant chemokine/chemokine receptor expression, increased baseline phagocytosis, and attenuated proliferation. Finally, monocyte‐macrophage differentiation was deregulated in NEAT1−/− bone marrow and blood. Finally, monocyte‐macrophage differentiation was deregulated in NEAT1−/− bone marrow and blood.^94^ MALAT1‐deficient ApoE−/− mice display atherosclerosis and their BMDMs responded to LPS show enhanced pro‐inflammatory cytokines expression including TNF and inducible NO synthase (NOS2).^93^ It is likely that the direct interactions between MALAT1 and NEAT1 through the enzymatically MALAT1‐derived mascRNA might promote the development of atherosclerosis. Knockdown of lncRNA Mirt1 attenuates acute MI injury which could be attributed to the reduced inflammatory macrophage infiltration through inhibition of the NF‐κB pathway.^95^ Recent studies have highlighted that circRNA may represent a potential new therapeutic target in cardiovascular disease^96^; however, their function and molecular mechanism correlated with macrophages remain largely unknown and await further detailed study. Inflammation and fibrosis are the major risks for heart failure with preserved ejection fraction (HFPEF) patients, evidenced by the increased M1 and M2 numbers in HFPEF, and the HFPEF patient‐derived sera could promote healthy donor monocytes into M2 macrophage.^97^ Neutrophils are another type of innate immune cell that is involved in cardiac repair after MI by polarizing macrophages toward a reparative M2 phenotype.^98^ Increased fibrosis was found in neutrophil‐depleted mice subjected to MI and the phenotype of macrophage can be changed by administration of neutrophil secretome or neutrophil gelatinase‐associated lipocalin.^98^ These experimental models and clinical successes have led to a macrophage‐centered view of antifibrotic approach in CF. Considering recent reports on the control of macrophage polarization by ncRNAs provided via the internal and external stimuli, various ncRNAs might be identified as candidate targets for therapeutic intervention in the CF microenvironment (Figure 6).

## 7 CONCLUSIONS AND PERSPECTIVE

As is known to all, the development and progression of fibrosis involve the interaction of distinct and overlapping mechanisms which orchestrate the roles and actions of multiple residents and recruited monocyte/macrophages. On the one hand, M1 macrophages initiate tissue inflammation that underlies the predominant and protective responses to tissue injury. On the other hand, prolonged inflammation promotes the maladaptive tissue remodeling and fibrosis process, which leads to chronic pathology, partially mediated by M2 macrophages. Accumulating evidence identifies multiple types of ncRNAs as key mechanistic regulators of persistent M1 and/or M2‐dependent tissue damage and fibrosis in a wide variety of organ systems.^99^ Distinct ncRNAs‐regulatory modalities may be required for effective reprogramming of macrophage polarization in the specific fibrotic conditions. For this purpose, there is an urgent need to improve our understanding of the internal connections between different ncRNAs and different organs. Additionally, studies in animal models did not fully reflect the identity of humans, additional confirmatory studies would be necessary for elucidating the specific ncRNAs‐mediated M1/M2 gene expression profiles and transcriptional regulatory pathways in humans. To date, most of the new macrophage‐centered strategies have been tested in animal or early clinical trials that are not sufficient to fully reflect the clinical values in fibrotic patients therapy. Thus, further large translational studies and clinical trials, based on the interplay between ncRNAs and macrophage polarization, could be a way to identify more efficiently promising treatments for fibrotic diseases.

## CONFLICT OF INTERESTS

The authors declare that there are no conflict of interests.

## AUTHOR CONTRIBUTIONS

DZ designed and planned the work, and revised the manuscript. YW and SW performed the literature search and interpretation, and manuscript drafting. JL and JJL revised the manuscript.

## ETHICS STATEMENT

The study was approved by the Institutional Review Board of Wannan Medical College, Wuhu, China. We certify that this manuscript is original and has not been published and will not be submitted elsewhere for publication while being considered by *Immunity, Inflammation and Disease*. No data, text, or theories by others are presented as if they were our own. The submission has been received explicitly from all co‐authors. And authors whose names appear on the submission have contributed sufficiently to the scientific work and, therefore, share collective responsibility and accountability for the results.
